# Developing STEP‐SE: A Qualitative Usability Study of a Novel Patient‐Reported Outcomes Tool for Managing Side Effects in Shared Decision‐Making for Schizophrenia Spectrum Disorder Care

**DOI:** 10.1111/hex.70019

**Published:** 2024-11-01

**Authors:** Alessandro Rodolico, Antonio Di Francesco, Pierfelice Cutrufelli, Irene Bighelli, Pasquale Caponnetto, Carmen Concerto, Davide Conti, Rosaria Furnari, Gabriele Leotta, Ludovico Mineo, Antonino Messina, Katharina Müller, Antonino Petralia, Maria Catena Quattropani, Spyridon Siafis, Stefan Leucht, Maria Salvina Signorelli

**Affiliations:** ^1^ Department of Clinical and Experimental Medicine Institute of Psychiatry, University of Catania Catania Italy; ^2^ Department of Psychiatry and Psychotherapy Technical University of Munich, TUM School of Medicine and Health Munich Germany; ^3^ Department of Educational Sciences Section of Psychology, University of Catania Catania Italy; ^4^ Center of Excellence for the Acceleration of Harm Reduction (COEHAR) University of Catania Catania Italy; ^5^ Darwin Technologies Catania Italy; ^6^ Department of Mental Health Provincial Health Authority of Enna Enna Italy; ^7^ kbo‐Isar‐Amper‐Klinikum München Munich Germany; ^8^ Oasi Research Institute ‐ IRCCS Troina Italy

**Keywords:** antipsychotics, digital decision aids, qualitative, schizophrenia, shared decision‐making, side effects, usability

## Abstract

**Background:**

Schizophrenia treatment with antipsychotics often results in side effects that impact adherence and quality of life. Managing these effects remains challenging, as it requires balancing efficacy and tolerability. The Schizophrenia Technological Evaluation of Patient Side Effects (STEP‐SE) app aims to aid side effects monitoring and management through shared decision‐making (SDM).

**Aim:**

This study aimed to evaluate the usability of the STEP‐SE app for patients and clinicians in managing antipsychotic side effects.

**Methods:**

Sixteen stable outpatients and 14 psychiatrists participated in semi‐structured interviews after using the STEP‐SE app. Questions explored ease of use, information clarity, user needs fulfilment, patient–clinician collaboration, treatment adherence improvement, patient empowerment and clinical utility. Data were analysed thematically.

**Results:**

Overall satisfaction with STEP‐SE was high. Both groups found that the tool improved patient involvement, provided reliable information to enhance therapeutic alliance, posed low risks of misunderstanding and had an intuitive interface. Patients felt more motivated and empowered. Clinicians appreciated guideline consistency. Preferences differed regarding data visualization formats.

**Discussion:**

STEP‐SE shows potential for aiding SDM on antipsychotic side effects. Patients gained motivation, and clinicians felt reassured. Refinements around mobile access, graphics and features could augment utility. Generalizability is limited given the stable patient sample.

**Conclusion:**

Preliminary findings suggest that STEP‐SE effectively engages patients, empowers them and supports clinicians in collaborative side effect management. Further testing with diverse user groups is warranted.

**Patient or Public Contribution:**

The current study was designed to gather patient and public feedback for the development of our decision aid tool, STEP‐SE. Participants interacted with the tool's prototype in interactive sessions, providing insights and identifying technical issues. Their feedback was crucial for enhancing the tool, with each suggestion and bug report carefully considered for future iterations. The participants’ contributions were key in optimizing STEP‐SE's features and ensuring its relevance and reliability. We thank all who shared their time and perspectives, significantly shaping the tool's user‐centred design.

## 1 Introduction

Schizophrenia is a complex chronic mental disorder, characterized by positive symptoms, such as delusions and hallucinations, and negative symptoms including reduced motivation. It is managed primarily through antipsychotic medications [1]. These medications are effective but often have significant side effects, affecting up to 75% of patients [2], that can impact treatment adherence [3] and quality of life [4]. Managing these side effects is a key psychiatric challenge. Minimal effective antipsychotic doses are recommended to reduce side effects, but increase relapse risk [5]. Additionally, although polypharmacy may result in more side effects than improvements in efficacy [6], there are specific circumstances where using multiple medications or higher dosages may be beneficial. In these cases, careful monitoring and clinical judgement are crucial to optimize treatment outcomes and minimize risks [7], ideally guided by evidence‐based approaches where digital health tools can play a crucial role [8].

Digital decision aids (DDAs) enable informed patient–clinician decisions by providing personalized, evidence‐based conditions and treatments [9]. Effective DDAs simplify data [10], support transparent shared decision‐making (SDM) processes [11] and facilitate the transition from paternalistic to participatory models.

The STEP‐SE (Schizophrenia Technological Evaluation of Patient Side Effects) app is a pivotal example of such a DDA. It utilizes a validated scale for side effects to collect comprehensive data, where patients report the frequency and distress level of each side effect experienced [12]. The software processes the patient‐reported information to generate an integrated overview of possible interventions, which is accessible to both the clinician and the patient. This synopsis facilitates a mutual understanding of the patient's current health status and guides subsequent treatment decisions. It includes several detailed sections: one that considers whether further diagnostic evaluation is necessary, another evaluating potential drug interactions that might be exacerbating side effects and a final section suggesting additional interventions. These might include lifestyle adjustments, supplementary therapies or changes in medication, all tailored to the patient's specific needs and situation. For a detailed description of STEP‐SE, please refer to Section 2.1.

STEP‐SE was developed after the implementation of the Shared Decision‐Making Assistant (SDMA), a DDA designed to improve the SDM process. SDMA operates as a web app that collects data on side effects that patients find intolerable when starting new treatments. It also tracks the previous use of antipsychotics, allowing patients and clinicians to avoid them when starting a new treatment. SDMA includes forest plots that compare efficacy and side effects, enabling clinicians and patients to make informed, collaborative decisions upon admission. This innovative tool is currently undergoing a cluster‐randomized trial to assess its effectiveness compared to standard practices for schizophrenia patients during acute treatment phases [13]. Although SDMA focuses on acute schizophrenia phases to enhance patients’ perceived involvement in decision‐making, STEP‐SE is tailored for stable patients managing antipsychotic side effects, providing current evidence on side effect management and promoting collaborative, non‐prescriptive decision‐making.

This study aims to showcase the STEP‐SE app as an advanced tool for schizophrenia management. It details the app's features, theoretical foundations and findings from usability tests and interviews with patients and clinicians. The data, analysed using qualitative framework analysis, focus on usability aspects such as ease of use, clear data presentation, user needs fulfilment, improved patient–physician collaboration, treatment adherence, patient empowerment and clinician utility.

## 2 Methods

STEP‐SE was developed according to the recommendations of the International Patient Decision Aid Standards (IPDASi) v4.0 (Appendix S1) [14]. This study was approved by the Internal Ethics Review Board of the Psychology Section of the Department of Education Sciences, complying with the guidelines provided by the Italian Association of Psychology (AIP) and its Ethical Committee, and meeting standard data privacy criteria (Prot. n° Ierb‐Edunict‐2023.06.08/03).

### 2.1 STEP‐SE Development

STEP‐SE is a web‐based application designed to manage antipsychotic‐induced side effects in patients with stable schizophrenia spectrum disorders and can be accessed through any web browser. It offers an interactive experience in which patients and clinicians engage in a synchronous exchange of information. The primary interactive feature of the app includes a self‐report digital questionnaire that patients use to input any side effects they experience from antipsychotic medications. This, combined with clinician data input covering the patient's clinical history and current medications, generates a summary of potential approaches for addressing the side effects. This summary is presented to both the clinician and the patient during their consultation, supporting an SDM process. Specifically for this project, STEP‐SE was configured for local storage on a Windows™ computer, facilitating offline data processing (detailed in Appendix S1). To safeguard data confidentiality, the app requires authenticated clinician access as the first step (Supporting Information S1: eAppendix img.1). The physician then enters the patient's demographic data and details of their current therapy, including both psychiatric and non‐psychiatric medications (Supporting Information S1: eAppendix img.2). In cases of polypharmacy, the physician identifies the primary antipsychotic medication (Supporting Information S1: eAppendix img.3) based on the highest dose equivalence according to the defined daily dose (DDD) method [15]. This selection is critical as the app's subsequent sections rely on the identified primary antipsychotic. In the patient‐specific section, users complete a digital Italian version of the Glasgow Antipsychotic Side‐Effect Scale (GASS) [12, 16], a standard tool for assessing side effects [17]. Patients then identify up to three distressing side effects, refining the tool's treatment suggestions (Supporting Information S1: eAppendix img.4). The app presents results in three subsections. The first subsection helps distinguish side effects from non‐antipsychotic determined factors that may require further evaluation, such as increased prolactin levels potentially due to a pituitary adenoma. The second subsection provides a link to the DrugBank website for comprehensive drug interaction checks (https://go.drugbank.com/drug-interaction-checker) [18]. The third subsection outlines strategies such as dose reduction, add‐on treatments, behavioural changes, timing adjustments or switching antipsychotics (Supporting Information S1: eAppendix img.5). These data align with English‐language clinical guidelines that meet or exceed the S2E evidence level as per the Association of Scientific Medical Societies in Germany (AWMF) model [19]. Specifically, it incorporates recommendations on antipsychotic‐induced side effects from British [20], Japanese [21], Scottish [22], American [23] and German [24] guidelines. The last subsection is formatted as a table, with intervention options and selected side effects as column headers. Each cell displays a checkmark or cross to indicate guideline recommendations for each intervention–side effect combination (Supporting Information S1: eAppendix img.6). The dose reduction page, used as needed according to guidelines, includes a graph estimating relapse risk from dose changes based on Leucht et al.'s dose–response meta‐analysis [25], using a risperidone dose‐equivalence slider to adjust and dynamically update relapse risk (Supporting Information S1: eAppendix img.7). The switch page displays forest plots from network meta‐analyses for visual treatment comparisons (Supporting Information S1: eAppendix img.8), sourced from studies on various side effects [26, 27, 28, 29, 30]. On the dose reduction page, dose equivalents are specific to each antipsychotic, whereas in the switch graph, the primary antipsychotic is used as the ‘control’ treatment for comparison. In response to initial interview feedback, where patients found the forest plot data on medication switching hard to interpret and clinicians recommended a simpler presentation method, we created a semi‐quantitative dynamic table to sort antipsychotics by their effectiveness on selected side effects. We also revised our questionnaire to assess preferences for this new format versus the original.

### 2.2 User‐Testing Study

We followed the COREQ guidelines for reporting qualitative research [31].

#### 2.2.1 Research Questions

Our study addressed the following research questions:

1. How STEP‐SE might facilitate SDM for patients with schizophrenia spectrum disorders and their healthcare providers?
2. In what ways STEP‐SE might enhance patient autonomy and involvement in managing antipsychotic medication side effects?
3. How do clinicians and patients perceive the usability and effectiveness of STEP‐SE in clinical practice?

#### 2.2.2 Personnel Characteristics and Relationship With Participants

All interviews were conducted by A.R., A.D.F. or P.Cu. A.R. is a specialist in psychiatry. A.D.F. and P.Cu. are medical doctors and third‐year psychiatric residents. All the interviewers have at least 1 year of experience with patients with schizophrenia. The training and supervision were provided by P.Ca., a researcher of clinical psychology and psychotherapist, with many years of experience in conducting and supervising qualitative studies in people with schizophrenia spectrum disorders. None of the interviewers were personally known to the patients, and there was no therapeutic relationship between the parties. The treating psychiatrists (L.M., C.C., R.F., A.M., M.S.S. and A.P.) presented the patients to the interviewers. Participants were not informed that the tool was developed by their interviewers, who were trained to remain neutral and did not reveal their connection to the tool. However, it is unknown whether participants deduced the relationship between the tool and the study team. However, the clinicians were selected by convenience sampling among the colleagues of A.R. In this case, these were aware of the fact that the tool was developed by the study authors. The sample size was pre‐established based on Creswell's guidelines [32], which suggest that qualitative phenomenological studies typically require between 5 and 25 participants.

#### 2.2.3 Theoretical Framework

We performed a thematic analysis based on semi‐structured interviews, which focused on several key areas of interest. These included participants’ attitudes towards SDM, the impact of a digital tool on managing side effects from antipsychotics and the tool's usability, assessed through Jakob Nielsen's dimensions of learnability, efficiency, memorability, errors and satisfaction [33]. Additionally, we examined the psychological effects associated with using this digital tool, ensuring a comprehensive evaluation of both its functional and emotional impacts on users. Consequently, although the selection of topics partially guided the content derived from our thematic analysis, we also made an effort to report comprehensively on the themes that emerged. We conducted individual semi‐structured interviews both with patients and clinicians. We decided to interview both specialists and residents in psychiatry to obtain perspectives from diverse generations of clinicians.

#### 2.2.4 Participant Selection and Setting

Participants were chosen using purposive sampling to match the tool's user profile, with criteria including adults (18+), outpatient care, confirmed schizophrenia spectrum disorder per DSM‐5‐TR, stable antipsychotic treatment for over 6 months, no acute positive symptoms (PANSS Positive Items ≤ 3), sufficient insight (g12 PANSS item ≤ 3) and study comprehension and consent capability. We excluded inpatients, as their condition typically indicates acute episodes. Clinicians, including both specialists and residents, were selected through face‐to‐face convenience sampling, aiming for diverse gender, age and experience. We aimed to achieve an equal distribution of age and sex among our participants, seeking to represent individuals with diverse demographic characteristics within the population of interest. The study involved 30 participants with no refusals to participate. Interviews with both groups took place at the University Hospital ‘Policlinico G. Rodolico—San Marco’ in Catania (Italy) and, for some clinicians, at their workplaces. Patient interviews also took place at the Oasi Regina Pacis mental health centre in Catania (Italy). The interviews were conducted from 12 June to 21 October 2023.

#### 2.2.5 Data Collection

After the presentation of the tool, both patients and clinicians had time to interact with and explore its features. This hands‐on experience was crucial for them to become more comfortable and proficient in its use, despite it being their first time managing the tool.

Interviews began with collecting sociodemographic data and administering questionnaires on device/Internet usage (patients) and attitudes towards decision aids/SDM tools (clinicians). While sharing common elements on usability, learning ease and information preferences, interviews differed for each group. Patient interviews explored their discussions with doctors regarding therapy side effects, and how the app influenced their health management and treatment adherence. For clinicians, interviews explored their use of the app in patient interactions, and its impact on the doctor–patient relationship and professional well‐being.

Details of the questions are given in Appendix S1. Interviews lasted 30–45 min. Each interview was recorded and initially transcribed with the OpenAI Whisper automatic speech recognition (ASR) model [34] used locally on a Mac computer. The recordings were never uploaded online. The authors consequently checked the correspondence of the transcript to the recordings, corrected errors or omissions and restructured the transcript in a question/answer form as much as possible, given the semi‐structured nature of the interview. We informed the participants that they could receive back the transcript on request. Considering our research's focused aim on STEP‐SE's usability, we concluded that the selected number of participants was adequate for achieving thematic sufficiency.

#### 2.2.6 Data Analysis

The interviewers (A.R., A.D.F. and P.Cu.) coded the data. The data analysis was conducted following the principles of thematic analysis as described by Braun and Clarke [35]. Initially, we repeatedly listened to recordings and annotated transcripts. Two independent evaluators then coded each transcript, highlighting key interview segments. After double coding each interview, the team reviewed and finalized codes, initially group‐coding 30% of the interviews to establish a framework. This framework guided the coding of remaining interviews, with new codes approved in team meetings. Themes emerged from converging codes within similar areas. Based on the structure of the interview five macro‐areas were pre‐defined: doctor‐patient relationship and involvement in decision‐making; interaction with technology and app usability; management of treatment and side effects; feedback and development of STEP‐SE; psychological impact of using STEP‐SE. Emerging themes were grouped within these areas, using Excel for synthesis. While emphasizing the predefined macro‐areas aligned with our core research objectives, we made concerted efforts to retain comprehensive information from the data, reporting as many emerged themes as possible. We prioritized themes that were most frequently mentioned, considering their importance in the interviews. Therefore, even if the most salient aspects were the most commented on, we did not discard those that appeared only a few times but were insightful to our investigation. To further validate our findings and enhance the study's robustness, we sought feedback from a subset of patients and clinicians on the results.

## 3 Results

### 3.1 Participants

We conducted 30 interviews with 16 patients and 14 clinicians. Detailed demographic data for these participants are provided in Supporting Information S1: eAppendix Tables 1 and 2. The clinicians comprised a diverse mix of specialists and residents. Specialists, averaging 50 years old with 5–35 years of experience, worked in hospitals or mental health centres. Residents, averaging 32 years, had 1–4 years of experience in intensive hospital training environments. Physicians managed an average of 22.64 schizophrenia patients monthly. Their experience with mental illnesses varied, with 35.71% highly experienced, another 35.71% moderately experienced and 28.57% less experienced. Most (92.86%) believed that decision aids enhance clinical decision quality, and 71.43% observed improvements in communication (Supporting Information S1: eAppendix Table 3). Patient participants, predominantly male (75%) with an average age of 40, mostly had high school education. They have been receiving specialist treatment for an average of 10 years, with many experiencing therapy changes in the past year. To assess their familiarity with digital devices, we asked patients to rate their average experience using a visual analogue scale. This scale ranged from 0, indicating no experience with digital devices, to 10, indicating a very good experience, with 5 representing an average experience. The mean score reported by the patients was 6.44 out of 10, suggesting a moderately high level of familiarity with digital devices. A significant 92.86% of participants reported awareness of their medication indications, reflecting a high level of engagement with their treatment regimen (Supporting Information S1: eAppendix Table 4).

### 3.2 Qualitative Analysis

The perspectives of the participants were consolidated into five distinct categories and multiple themes (refer to Table 1). For participants' quotes about the first two category themes, please refer to Supporting Information S1: eAppendix Table 5. A summary of the feedback regarding UX/UI, data visualization and new features is provided in the following sections.

**Table 1:** Participants' perspectives organized in categories and relative themes.

Category	Themes (clinicians)	Themes (patients)
Dynamics of the doctor–patient relationship and involvement in decisions	**—** Clinician's prescriptive approach and paternalistic tendencies **—** Patient needs and engagement in decision‐making post‐stabilization **—** Importance of patient insight in shared decision‐making **—** Concerns about information overload and misinterpretation **—** Insights of patients' attitudes on independently searched information	**—** Diverse attitudes towards patient involvement in decision‐making **—** Diverse attitudes towards patient communication with clinicians **—** Strong deference to medical authority among patients desiring involvement **—** Patients' satisfaction with involvement despite limited communication
The app in the management of treatment and side effects	**—** App as a tool for enhancing patient involvement and therapeutic choice‐making **—** Efficiency in identifying side effects and formulating treatment strategies **—** Appreciation of the app as a reliable source of information and SDM tool **—** Concerns about potential information overload and misinterpretation risks	**—** Varied views on the utility of STEP‐SE for improving trust in medical decisions **—** App as a potential part of therapy and its role in enhancing the therapeutic alliance **—** App's role in patient autonomy and motivation **—** Concerns about potential misunderstandings arising from app use
Feedback: UX/UI	**—** Overall satisfaction with the app's functionalities **—** Mixed confidence in using the app after a period of non‐use **—** Suggestions for improvement in app operability and user interface design	**—** Positive user interface (UI) and usability experience **—** Simplicity and intuitiveness as commonly praised features **—** Challenges with navigation and complexity of exploring alternative medications **—** Suggestions for UI improvements including colour coding and larger text
Feedback: Data visualization (graphs vs. tables)	**—** Differing opinions on the usefulness of graphical representations vs. descriptive tables **—** Preference for descriptive tables for better visualization and orientation **—** Value in retaining both formats for their respective strengths	**—** Preference for graphical representations for understanding medication switch **—** Favouring descriptive tables for their clear presentation of switch data
Feedback: New features	**—** Proposals for new features to enhance therapeutic decision‐making **—** Need for more detailed information on medication switches and relapses ‐ Request for inclusion of depot formulations and biometric parameters **—** Call for a mobile version to increase accessibility and practicality	**—** Desire for a journaling feature and reporting concerns to drug regulatory agencies **—** Request for a mobile version of the tool for enhanced accessibility

#### 3.2.1 Dynamics of the Doctor–Patient Relationship and Involvement in Decisions

##### 3.2.1.1. Patients’ Perspectives

The thematic analysis revealed diverse attitudes towards patient involvement in clinical decision‐making. Six patients showed an active approach, moving from passively following doctors’ prescriptions to engaging in dialogue and making joint medication decisions with their providers. This shift towards active participation reflected a preference for collaborative decision‐making with clinicians, a sentiment shared by others who highlighted the crucial role of patient involvement in therapeutic choices during medical consultations. This appreciation for involvement was evident as participants expressed a desire to be part of the decision‐making process. Although many patients desired involvement, there was a notable deference to medical authority.

Building on this understanding of varied patient engagement, another key theme identified from the interviews was the differing attitudes towards communication with clinicians. Multiple patients reported positive experiences when discussing potential therapies, feeling acknowledged and comforted by their doctors. The personal care received during these discussions was highly valued, enhancing the sense of being cared for rather than just treated as a number. However, a contrasting perspective emerged as well. Some patients reported seldom discussing independently found health information with their doctors, with responses ranging from ‘rarely’ to ‘almost never’. Despite this limited dialogue, a subset of those expressed contentment with their involvement in treatment decisions. This suggests that the need for more in‐depth communication might sometimes be overlooked in patients who do not actively seek it but nonetheless appreciate being involved in their healthcare.

##### 3.2.1.2. Clinicians’ Perspectives

Three themes that arose in this category from clinicians were clinician's prescriptive approach, how patient needs are addressed and patients’ engagement outside of direct clinical interactions.

Clinicians with a paternalistic approach, particularly in acute psychiatric settings, often had minimal patient discussions about treatment options. It's also noted that although there might be internal group discussions among colleagues about future treatments, these discussions were generally not shared with patients. However, some clinicians preferred to involve the patient in decision‐making only after stabilization. In emergency and urgent care settings, discussions were initiated as part of the care plan from admission, with an acknowledgement that success in involving patients depends on their awareness. For patients with severe schizophrenia, the focus was initially on stabilization. Once stabilized, these patients generally responded well to discussions about treatment options. The importance of patient insight is emphasized, highlighting that when patients show some insight and willingness, clinicians strive to involve them more in decisions, allowing them to describe their experiences with the therapy and collaboratively understand the risks and benefits of potential changes.

In debating about SDM, multiple clinicians emphasized not only the importance of involving patients and their families but also how this can strengthen the therapeutic alliance. However, concerns are raised about sharing too much information, as it might lead to non‐acceptance of the therapy or even a hidden desire to discontinue it.

Clinicians commonly observed patients discussing online health information, yet it was often noted that such information was frequently irrelevant. Age‐related nuances in how patients handle online information had also been discussed, with younger patients more likely to discuss their findings due to clinician inquiries, whereas older patients might rely on word of mouth or be hesitant to discuss their research unless prompted. A different perspective suggested a selective disclosure among patients who may not share all the concerns they have developed from online sources.

#### 3.2.2 The App in the Management of Treatment and Side Effects

##### 3.2.2.1. Patients’ Perspectives

Patients expressed varied opinions on STEP‐SE's utility. Some believed that it could enhance trust in medical decisions by making the decision‐making process easier for doctors and improving compliance when used collaboratively between doctors and patients. They pointed out that the app provides clear comparisons between medications, potentially reducing the need to trial numerous drugs. Others appreciated its role in facilitating doctor–patient interaction, which could strengthen the therapeutic alliance. However, it was noted that the app's effectiveness might be limited if the doctor–patient alliance is not strong.

The app's impact on patient autonomy and motivation emerged as a key theme. Several patients found the app beneficial for preparing for doctor visits and increasing awareness of side effects. Many found the app motivating and helpful for treatment commitment.

Conversely, some patients were sceptical about the app's ability to improve autonomy and motivation, highlighting the need for doctor approval in health decisions and questioning true autonomy.

Concerns were also noted about potential misunderstandings due to the app's use. Although most participants did not anticipate issues, there were concerns about the possibility of conflicts between the app's suggestions and medical advice, such as recommendations on medication dosages that might contradict what a psychiatrist advises.

##### 3.2.2.2. Clinicians’ Perspectives

Clinicians recognized the STEP‐SE app's dual role: enhancing patient involvement in therapeutic decisions and assisting clinicians in making therapeutic choices. The app was valued for its efficiency in identifying side effects and formulating treatment strategies. It was noted for its potential impact on prescribing practices, with some clinicians open to adjusting their methods based on the app's recommendations if they observed discrepancies with their own experience. Furthermore, clinicians viewed the app as a reliable source for alternative treatment options and believed it could streamline their workflow, save time and enhance patient engagement in therapy management, indicating its transformative potential in clinical settings.

The app was also valued as an SDM tool. Some doctors emphasized the importance of clear, scientifically grounded information that both doctor and patient can review together, which aids in building trust by providing patients with a clear guide and reassuring them of the scientific basis of their treatment.

Some clinicians found that STEP‐SE facilitates the doctor–patient relationship by aiding in medication direction and enhancing the therapeutic alliance. Features such as linking to DrugBank for checking drug–drug interactions were particularly valued for their ability to involve patients more directly and increase mutual trust.

Most of the clinicians also discussed the psychological benefits of using the app, feeling more confident and secure in their practice due to its alignment with verified guidelines. However, concerns were raised about potential information overload for patients and the possibility of content that may alarm patients unnecessarily. Risks of misinterpretation or excessive reliance on the app's suggestions for medication changes were also pointed out, with a cautious approach recommended for using the app with patients well known to the clinician, where shared decisions can truly improve clinical outcomes without the risk of setbacks.

Overall, although most of the clinicians were not concerned about misunderstandings with the app, there is a consensus on the importance of balancing its use with clinical judgement and maintaining the integrity of the doctor–patient relationship.

#### 3.2.3 Feedback: UX/UI

##### 3.2.3.1. Patients’ Perspectives

Patients reported a positive user interface (UI) and usability experience, noting the app's simplicity, intuitive design and clear initial instructions.

Favourite aspects of the app included its simplicity, intuitiveness and colourful design. The questionnaire section was also favoured for enhancing patient–doctor interaction. Most users were satisfied with the design, finding it functional and appropriate; however, some noted challenges with navigation and the complexity in exploring alternative medications.

Most patients found the app user‐friendly and memorable, feeling confident in their ability to use it after periods of non‐use. However, a few struggled with concentration and memorization, finding it hard to learn and retain information.

Some patients suggested UI improvements such as more vibrant colours, colour coding for drugs to enhance visual distinctiveness and larger text for better readability.

##### 3.2.3.2. Clinicians’ Perspectives

Clinicians were generally satisfied with the STEP‐SE app's functionalities, appreciating its simplicity, information richness and quick access to important functions. However, some noted difficulties with operational clarity.

Most clinicians felt confident using the app after periods of non‐use, although some anticipated needing re‐familiarization. Challenges were noted in navigating the app, especially for less tech‐savvy users and in time‐limited public healthcare settings.

Feedback on the UI was positive, with the app being intuitive yet some elements causing confusion. Suggestions for improvement included better design uniformity and more seamless integration of drug interaction information to avoid reliance on external websites.

Most clinicians reported no software bugs, with only minor start‐up malfunctions mentioned, which were quickly resolved.

#### 3.2.4 Feedback: Data Visualization (Graphs vs. Tables)

##### 3.2.4.1. Patients’ Perspectives

One‐third of patients preferred graphical representations for understanding medication changes, finding them straightforward and reminiscent of school lessons. However, others favoured descriptive tables for their clarity and ease of comparison, especially suitable for those less comfortable with mathematical concepts.

##### 3.2.4.2. Clinicians’ Perspectives

Some clinicians appreciated the straightforwardness of graphical representations, but most found them less intuitive for medication switches and suggested enhancing their user‐friendliness. Descriptive tables were preferred for their ease of understanding and visual clarity. Clinicians recommended maintaining both formats, recognizing the visual impact of forest plots and the detailed information provided by tables.

#### 3.2.5 Feedback: New Features

##### 3.2.5.1. Patients’ Perspectives

Patients suggested new features for the app: a journaling function for daily mental state entries, the inclusion of drug brand names for better familiarity, a direct reporting feature for adverse reactions and a mobile version to improve accessibility and convenience.

##### 3.2.5.2. Clinicians’ Perspectives

Clinicians recommended several enhancements: a bookmarking feature for the therapeutic options that both clinicians and patients considered, more detailed relapse information, clearer guidance on medication switches, inclusion of depot formulations, expanded content on medication combinations, capability to gather biometric parameters and a mobile version to improve accessibility and practicality.

## 4 Discussion

In our study, we introduced STEP‐SE, a novel SDM tool, and explored its use in a qualitative study with patients and clinicians. The data revealed varied clinician approaches to SDM, ranging from paternalistic to patient‐inclusive styles, particularly post‐stabilization. Patients showed a desire for involvement yet also displayed passivity and reliance on clinician decisions. Most of the participants reported awareness of their medication indications, demonstrating a high level of competence with their treatment. This awareness likely enhances their involvement in SDM processes facilitated by electronic decision aids. Understanding one's treatment not only fosters a sense of agency but may also enhance the effectiveness of decision aids in supporting more informed and personalized treatment choices. While primarily an SDM aid, STEP‐SE was valued for its broader clinical management benefits. Both groups noted a low risk of misunderstandings; however, such issues might not be often highlighted in the qualitative analysis due to the close interactions between participants and interviewers. Nonetheless, both patients and clinicians raised concerns about the potential misuse of the app. The UX/UI experience was positively received, with minimal requests for interface modifications. This aligns with Nielsen and Molich's usability standards [36], particularly in terms of learnability, efficiency and memorability. Patients felt more involved in therapy, and clinicians valued the tool's guideline adherence.

We presented data on antipsychotic switching through both forest plots and a tabular format to assess user preferences for data visualization. Although some participants were attracted to the forest plots, there were concerns about readability. This feedback highlights a crucial area for future development, suggesting a need for refined or alternative graphical representations to enhance the clarity and accessibility of data for both clinicians and patients. Regarding new features, the initial plans to include brand drug names and depot formulations were not realized in the current version. Patients’ requests for a journaling feature and other suggestions were outside the initial scope of the app but provided valuable insights for future development. Key improvements proposed included the ability to bookmark relevant options, detailed guidance on switching antipsychotics and a mobile version of the tool. Originally intended for inpatient settings, the potential for broader application was recognized by both clinicians and patients, suggesting the development of a mobile version as a priority for expanding the tool's utility in various contexts.

The study of STEP‐SE faced several limitations that should be considered. Methodologically, the assessment of the tool's memorability and the participants’ comprehension of the graphs were not thoroughly evaluated. Additionally, the semi‐structured interview format may have restricted the emergence of insights that a more open‐ended approach, such as focus groups, could have elicited. To mitigate potential distress, we predefined a maximum duration of 1 h for each visit to complete the STEP‐SE questionnaire based on preliminary feedback indicating that longer sessions could lead to patient discomfort or fatigue. In instances where the maximum time was reached, particularly for two patients who exhibited signs of fatigue, we scheduled a second meeting to ensure their comfort and gather comprehensive feedback on their views regarding SDM and DDAs. This approach, although necessary to prevent distress, may have constrained the depth of feedback obtained during the interviews. The generalizability of the findings is also limited because the study only involved stable patients, which prevents us from extending our results to acute patients. Moreover, the unequal distribution of sex among patients and the skewed age distribution among clinicians due to the inclusion of residents may have influenced the findings, limiting the representativeness of the results. Additionally, the experiences of depot patients were not distinctly analysed as their depot treatments were converted to oral formulations for the study, and the views captured may not fully represent the broader population of patients with schizophrenia spectrum disorders. Existing literature indicates that patients receiving depot antipsychotics may experience a heightened sense of coercion [37] and may encounter greater levels of stigma [38] distinguishing them from patients on oral medication regimens. Furthermore, the responses of psychiatrists may have been subject to interviewer bias, reflecting a tendency to portray themselves as less paternalistic and more collaborative in decision‐making than they might actually be, perhaps due to prevailing cultural norms valuing empathy and egalitarianism in patient care [39]. For the patient interviews, although efforts were made to minimize bias by ensuring that interviewers did not disclose their involvement in developing the tool, we did not explicitly assess the participants’ perceptions of the interviewers’ relationship with the tool. Therefore, we cannot exclude the possibility of interviewer involvement bias. Lastly, the current study design does not allow for a detailed measurement of patients’ perceived involvement in medication decisions. A longitudinal study would be more suitable for assessing this aspect comprehensively, providing insights into the sustained use and impact of the tool over time. These factors collectively highlight areas for careful consideration in interpreting the study results and suggest directions for future research.

To sum up, the app's core functionality involved gathering patient‐reported data through patient‐reported outcome measures, enabling the generation of personalized treatment recommendations in collaboration with clinicians. Our analysis revealed insightful findings regarding the app's effectiveness and user experience. Users reported a high degree of satisfaction with the app's interface and found the information provided on managing side effects to be comprehensive and easily understandable. This aligns with the broader trend of digital health tools enhancing patient empowerment, as documented in various studies [40]. Additionally, the app facilitated improved communication between patients and their healthcare providers, underscoring its potential as a valuable tool in the patient‐centred healthcare model.

## Author Contributions

**Alessandro Rodolico:** conceptualization, methodology, formal analysis, data curation, writing–original draft, writing–review and editing, investigation, project administration. **Antonio Di Francesco:** methodology, formal analysis, data curation, writing–original draft, investigation. **Pierfelice Cutrufelli:** methodology, data curation, writing–original draft, investigation. **Irene Bighelli:** conceptualization, writing–review and editing. **Pasquale Caponnetto:** methodology, writing–review and editing. **Carmen Concerto:** resources, writing–review and editing. **Davide Conti:** data curation, visualization. **Rosaria Furnari:** resources. **Gabriele Leotta:** software. **Ludovico Mineo:** resources. **Antonino Messina:** resources. **Katharina Müller:** methodology, writing–review and editing. **Antonino Petralia:** resources. **Maria Catena Quattropani:** writing–review and editing. **Spyridon Siafis:** conceptualization, writing–review and editing. **Stefan Leucht:** conceptualization, writing–review and editing, supervision. **Maria Salvina Signorelli:** supervision, writing–review and editing.

## Ethics Statement

This study was approved by the Internal Ethics Review Board of Psychology Research (IERB; Prot. n° Ierb‐Edunict‐2023.06.08/03).

## Consent

In accordance with the Declaration of Helsinki, both patients and clinicians have given informed consent for participation in this study. All data are confidential and used in compliance with ethical guidelines.

## Conflicts of Interest

P.Ca. has been affiliated with the CoEHAR since December 2019 in a pro bono role. He is a coauthor of a protocol paper supported by an Investigator‐Initiated Study award programme established by Philip Morris International in 2017. G.L. is an employee of Darwin Technologies, a company specializing in software and website development, including projects such as STEP‐SE for various clients. A.P. has been a consultant or a speaker or has received research grants from Allergan, Angelini, Janssen, Lundbeck and Otsuka. M.S.S. reports personal fees from Lundbeck and Angelini. S.L. has received honoraria for consultancy or advisory work or lectures from Angelini, Böhringer Ingelheim, Geodon Richter, Janssen, Johnson&Johnson, Lundbeck, LTS Lohmann, MSD, Otsuka, Recordati, SanofiAventis, Sandoz, Sunovion, TEVA, ROVI, EISAI and Medichem.

## Supporting information

Supporting information.

## Data Availability

The data that support the findings of this study are available from the corresponding author upon reasonable request.
