# Supplementary material for: Developing STEP‐SE: A Qualitative Usability Study of a Novel Patient‐Reported Outcomes Tool for Managing Side Effects in Shared Decision‐Making for Schizophrenia Spectrum Disorder Care
Source: Health Expect. 2024 Nov 1;27(6):e70019. doi: 10.1111/hex.70019 (PMC11528129; doi:10.1111/hex.70019)
Supplement: Supplementary file 1 — Supporting information. [file HEX-27-e70019-s001.docx]

**Developing STEP-SE: A Qualitative Usability Study of a Novel Patient-Reported Outcomes Tool for Managing Side Effects in Shared Decision-Making in Schizophrenia Care**

**eAppendix**

**Summary**

[International Patient Decision Aid Standards (IPDASi) v4.0 Checklist 2](#_Toc165735443)

[Technical description of the offline functioning of the app 9](#_Toc165735444)

[eAppendix img.1 10](#_Toc165735445)

[eAppendix img.2 11](#_Toc165735446)

[eAppendix img.3 12](#_Toc165735447)

[eAppendix img.4 13](#_Toc165735448)

[eAppendix img.5 14](#_Toc165735449)

[eAppendix img.6 14](#_Toc165735450)

[eAppendix img.7 15](#_Toc165735451)

[eAppendix img.8 16](#_Toc165735452)

[eAppendix Table 1 – Demographic characteristics of Physicians 17](#_Toc165735453)

[eAppendix Table 2 – Demographic charateristics of Patients 17](#_Toc165735454)

[eAppendix Table 3 – Physician’s Experience in Treating Mental and Psychotic Disorders and Opinions on the Use of Decision Aid Tools 18](#_Toc165735455)

[eAppendix Table 4 – Patients’ Personal Use of Digital Devices and Internet and Knowledge of Medication Indications 19](#_Toc165735456)

[eAppendix Table 5 – Participants relevant quotes extracted from the semi-structured interviews 19](#_Toc165735457)

[Semi-structured interview – Patients 21](#_Toc165735458)

[Semi-structured interview – Clinicians 22](#_Toc165735459)

# International Patient Decision Aid Standards (IPDASi) v4.0 Checklist

| **IPDAS dimensions** | **Qualifying criteria** | | **Certifying criteria** | | **Quality criteria** | |
| --- | --- | --- | --- | --- | --- | --- |
|  | Item | Comment | Item | Comment | Item | Comment |
| **Information** | *1. The patient decision aid describes the health condition or problem (treatment, procedure, or investigation) for which the index decision is required. (4.84)* | YES: The patient decision aid thoroughly describes side effects, which are compiled by the application itself. It also delineates their association with antipsychotic medications by presenting actual data | *1. The patient decision aid shows the negative and positive features of options with equal detail (e.g., using similar fonts, sequence, presentation of statistical information). (4.54)* | YES: It consistently maintains uniformity in graphical depiction, presentation style, and font choices throughout the application | *1. The patient decision aid describes the natural course of the health condition or problem, if no action is taken (when appropriate). (4.44)* | NA: The application is specifically designed to address immediate, present distress that is already actualized and definite. |
|  | *2. The patient decision aid explicitly states the decision that needs to be considered (index decision). (4.66)* | YES: It focuses explicitly on side effect management strategy choice |  | | *2. The patient decision aid makes it possible to compare the positive and negative features of the available options. (4.21)* | YES: It facilitates comparison of available options by presenting data that contrasts the risks and benefits associated with each choice |
|  | *3. The patient decision aid describes the options available for the index decision. (4.99)* | YES: It clearly outline the choices as between add-on medication, switching medication, or reducing medication dosage. |  |  |  | |
|  | *4. The patient decision aid describes the positive features (benefits or advantages) of each option. (4.80)* | YES: It effectively present the positive features of each option, with a clear presentation of their relative efficacy in reducing the burden of side effects, |  |  |  |  |
|  | *5. The patient decision aid describes the negative features (harms, side effects, or disadvantages) of each option. (4.89)* | YES: It comprehensively details the negative features of each option, specifically presenting the risk of relapse associated with the reduction or switching of medications. |  |  |  |  |
| **Probabilities** |  | |  | | *3. The patient decision aid provides information about outcome probabilities associated with the options (i.e., the likely consequences of decisions). (4.52)* | YES: It provides information on outcome probabilities associated with the options, detailing the likelihood of side effect manifestation or relapse risk |
|  |  |  |  |  | *4. The patient decision aid specifies the defined group (reference class) of patients for whom the outcome probabilities apply. (4.33)* | YES: It specifies that the outcome probabilities are based on data referring to patients within the schizophrenia spectrum treated with antipsychotics, clearly defining the reference class to which these probabilities apply. |
|  |  |  |  |  | *5. The patient decision aid specifies the event rates for the outcome probabilities (4.23)* | YES: It includes forest plots and tabular descriptions that report data using Relative Risk |
|  |  |  |  |  | *6. The patient decision aid allows the user to compare outcome probabilities across options using the same time period (when feasible).*  *(3.84) Amended to include comparison, and ‘when feasible’’ added.* | YES: outcome probabilities across different options are reported within the same timeframe |
|  |  |  |  |  | *7. The patient decision aid allows the user to compare outcome probabilities across options using the same denominator (when feasible). (4.46)* | YES: It facilitates the comparison of outcome probabilities across different options using a consistent denominator, employing relative measures with placebo as the reference |
|  |  |  |  |  | *8. The patient decision aid provides more than 1 way of viewing the probabilities (e.g., words, numbers, and diagrams). (3.43)* | YES: It offers multiple methods for viewing probabilities, including table representations, forest plots, and survival curves, thereby catering to diverse preferences for information processing. |
| **Values** | 6. The patient decision aid describes what it is like to experience the consequences of the options (e.g., physical, psychological, social) | NA: The focus of the decision aid is on the relief of side effects. |  | | *9. The patient decision aid asks patients to think about which positive and negative features of the options matter most to them (implicitly or explicitly).* | YES: patients are prompted to distinguish between side effects that are stressful and those that are not, and in identifying and prioritizing up to three side effects that are most significant for immediate management |
| **Guidance** |  | |  | | *10. The patient decision aid provides a step-by step way to make a decision. (2.97)* | YES: The SDM-assistant offers a multi-step process that begins with patients identifying their experienced side effects and categorizing them based on stress level. Subsequently, patients choose three side effects to manage, and the assistant proposes management strategies for each, guiding the patient step by step until a decision is reached. |
|  |  |  |  |  | *11. The patient decision aid includes tools like worksheets or lists of questions to use when discussing options with a practitioner. (3.17)* | YES: It provides synthetic representations of data, functioning as tools that patients can utilize when discussing options with a practitioner. |
| **Development** |  | |  | | *12. The development process included a needs assessment with clients or patients. (4.51)* | YES: As part of the development process, interviews were conducted with patients |
|  |  |  |  |  | *13. The development process included a needs assessment with health professionals. (4.07)* | YES: As part of the development process, interviews were conducted with health professionals |
|  |  |  |  |  | *14. The development process included review by clients/patients not involved in producing the decision support intervention. (3/71)* | YES: The application underwent evaluation by patients who were not part of the production team, external to the development process. |
|  |  |  |  |  | *15. Τhe development process included review by professionals not involved in producing the decision support intervention. (3.54)* | YES: The application was subject to evaluation by psychiatrists who were not involved in its production, ensuring an independent review by professionals external to the development process. |
|  |  |  |  |  | *16. The patient decision aid was field tested with patients who were facing the decision. (4.56)* | NO |
|  |  |  |  |  | *17. The patient decision aid was field tested with practitioners who counsel patients who face the decision. (4.56)* | NO |
| **Evidence** |  | | *2. The patient decision aid (or associated documentation) provides citations to the evidence selected. (3.85) ‘‘Studies’’ changed tο ‘‘evidence.’’* | No (Planned for future instances of the application) | *18. The patient decision aid (or associated documentation) describes how research evidence was selected or synthesized. (3.49)* | NO (Planned for future instances of the application) |
|  |  |  | *3. The patient decision aid (or associated documentation) provides a production or publication date. (4.02)* | YES: The tool includes the production date and version number, ensuring users are aware of its currency and iteration | *19. The patient decision aid (or associated documentation) describes the quality of the research evidence used. (3.77)* | NO (Planned for future instances of the application) |
|  |  |  | *4. The patient decision aid (or associated documentation) provides information about the update policy. (2.99)* | NO (Planned for future instances of the application) |  | |
|  |  |  | *5. The patient decision aid provides information about the levels of uncertainty around event or outcome probabilities (e.g., by giving a range or by using phases such as ‘‘our best estimate is*  *. . .’’). (3.53)* | YES: The patient decision aid presents the Relative risk of each event along with CI |  |  |
| **Disclosure** |  | | *6. The patient decision aid (or associated documentation) provides information about the funding source used for development. (4.39)* | YES: funding is reported | *20. The patient decision aid includes authors’/ developers’ credentials or qualifications. (3.51)* | YES: The credentials and qualifications of the authors and developers are included |
| **Plain language** |  | |  | | *21. The patient decision aid (or associated documentation) reports readability levels (using 1 or more of the available scales). (3.06)* | NO |
| **Evaluation** |  | |  |  | *22. There is evidence that the patient decision aid improves the match between the preferences of the informed patient and the option that is chosen. (3.44)* | NA: not tested yet |
|  |  |  |  | | *23. There is evidence that the patient decision aid helps patients improve their knowledge about options’ features. (3.67)* | NA: not tested yet |
| **Test** |  | | *7. The patient decision aid describes what the test is designed to measure. (4.90)* | YES: The questionnaire within the patient decision aid is explicitly designed to gauge the experience of side effects | *24. The patient decision aid includes information about the chances of having a true-positive test result.(4.74)* | NA |
|  |  |  | *8. If the test detects the condition or problem, the patient decision aid describes the next steps typically taken. (4.67)* | YES: Upon detection of a condition or problem, the application explicitly outlines the subsequent steps for side effects management, including options such as add-on medication, switching medication, or reducing dosage. | *25. The patient decision aid includes information about the chances of having a true-negative test result. (4.73)* | NA |
|  |  |  | *9. The patient decision aid describes the next steps if the condition or problem is not detected. (4.28)* | NA | *26. The patient decision aid includes information about the chances of having a false-positive test result. (4.77)* | NA |
|  |  |  | *10. The patient decision aid has information about the consequences of detecting the condition or disease that would never have caused problems if screening had not been done (lead time bias). (4.56)* | NA | *27. The patient decision aid includes information about the chances of having a false-negative test result. (4.78)* | NA |
|  |  |  |  | | *28. The patient decision aid describes the chances the disease is detected with and without the use of the test. (4.52)* | NA |

# Technical description of the offline functioning of the app

The web application STEP-SE was developed in a local development environment using tools provided by XAMPP, which includes the Apache web server and the MySQL database management system. This combination of tools made it possible to create and test the application directly on the developer's computer, providing a secure and controlled environment for development. The first step in developing STEP-SE was to configure XAMPP on the developer's system. This provided a local platform where Apache acted as a web server, allowing developers to run and test the application in isolation from the public internet. This approach ensured that any changes or updates could be tested in real-time without affecting an existing production environment. Simultaneously, a MySQL database was created and configured within XAMPP. The database was designed to meet the specific needs of STEP-SE, storing and managing crucial data such as user information, application settings, and other relevant information. MySQL was chosen for its reliability and ease of use, which are essential for a modern web application. The development of the user interface and backend logic of STEP-SE was done using scripting languages like PHP, which is well-supported by XAMPP. PHP allowed developers to write code that interacted dynamically with the MySQL database, performing operations such as data retrieval, insertion, and updating in response to user actions. In the day-to-day operation of STEP-SE, when a user accesses the application through a web browser, requests are sent to the Apache server. Apache processes these requests and forwards them to the appropriate PHP scripts. These scripts then communicate with the MySQL database to perform the required operations, which may include retrieving specific data or updating the database based on user interactions. One of the most advantageous aspects of using XAMPP for the development of STEP-SE was the ability to test the application in an environment that closely mimics a real web server. This allowed developers to conduct thorough testing, identify and efficiently resolve issues (debugging), ensuring that the application was robust and functional before being deployed in a production environment. In conclusion, developing STEP-SE through XAMPP provided developers with an integrated and flexible environment. The combined use of Apache and MySQL facilitated a development process that allowed developers to build a complex, secure, and functional web application tailored to the specific project's needs.

# eAppendix img.1 - App's Landing Page - Dual Access Interface


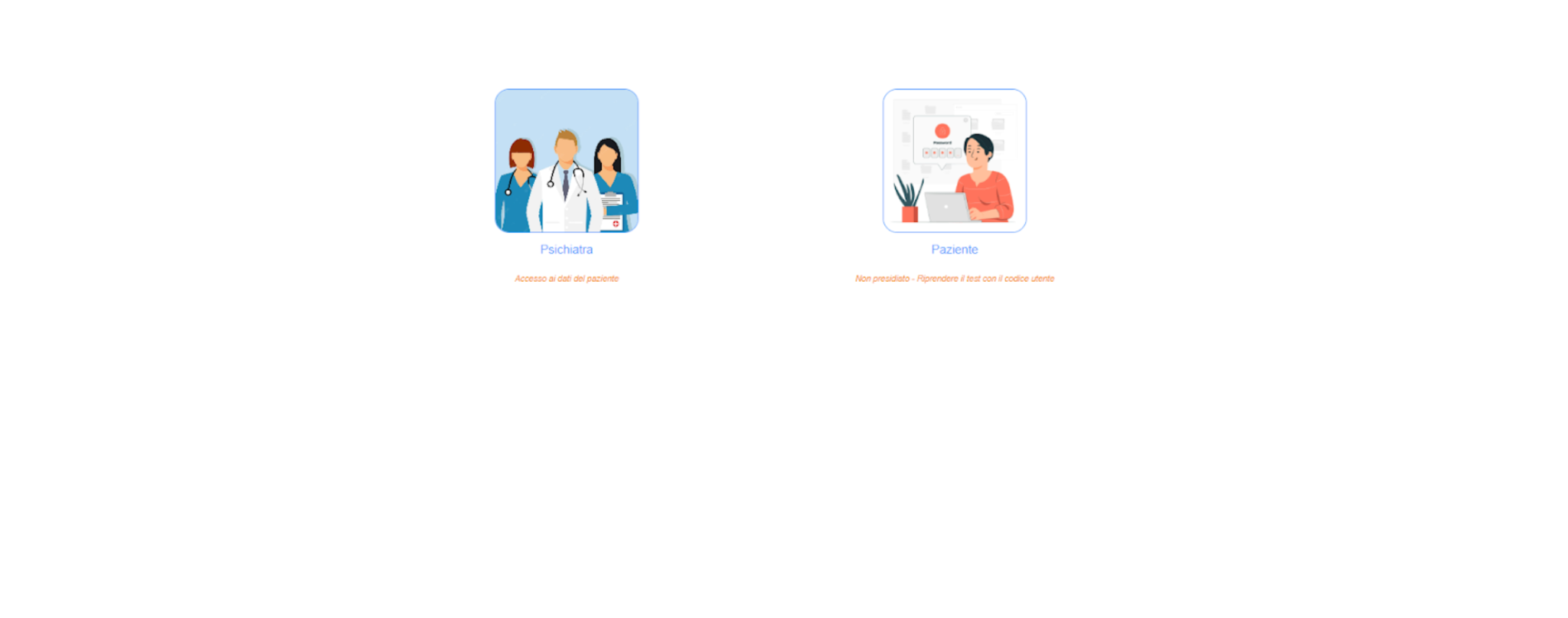
The landing page of the app features two distinct access points: 'Psichiatra' (Psychiatrist) on the left and 'Paziente' (Patient) on the right. The psychiatrist section allows clinicians to access patient data, while the patient section enables users to review their records or take tests using their unique user code.

# eAppendix img.2 - Medication Input Interface


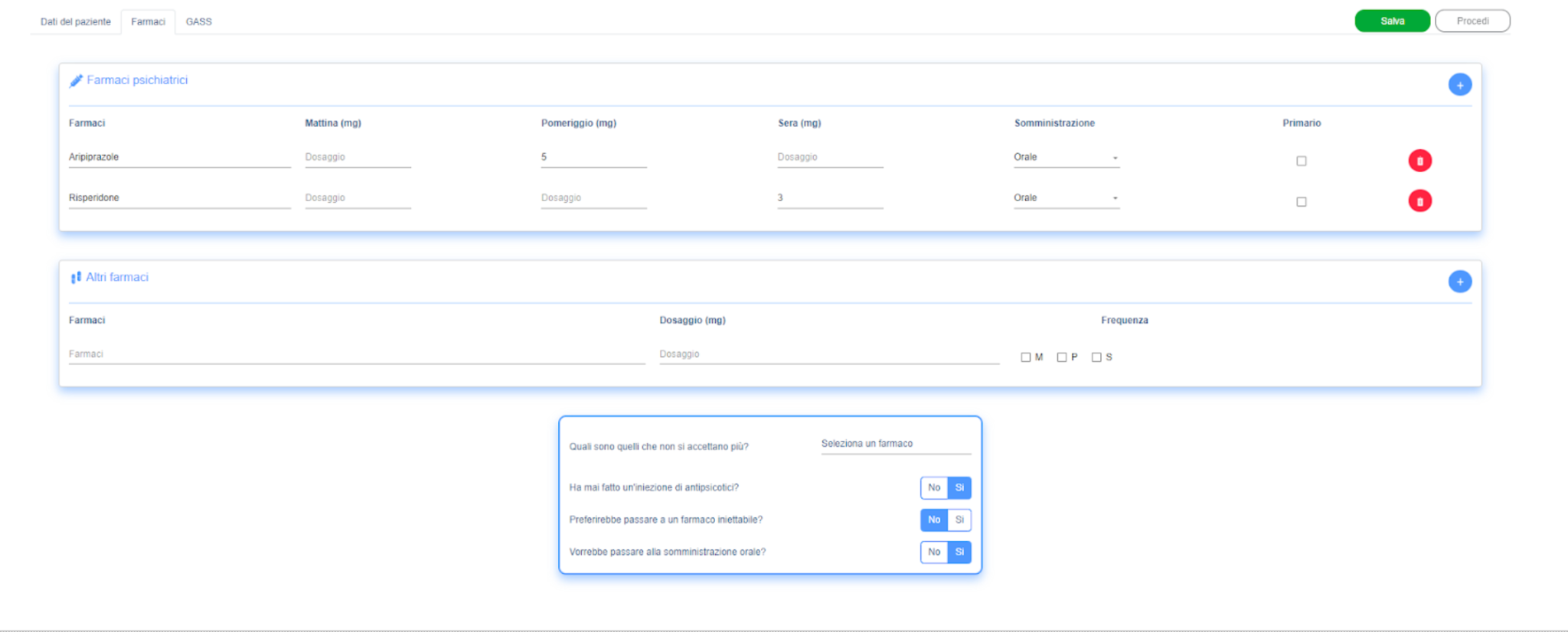


This page allows clinicians to input detailed patient medication data. The top section, 'Farmaci psichiatrici' (Psychiatric Medications), enables entry of antipsychotics with dosages for morning, afternoon, and evening, administration route, and a checkbox to indicate the primary antipsychotic. The middle section, 'Altri farmaci' (Other Medications), allows input of non-psychiatric drugs with dosage and frequency. The bottom section features four key questions about medication preferences and history, including undesired medications, experience with long-acting injectables, and preferences for switching to injectable or oral medications.

# eAppendix img.3 - Medication Input Interface (with checkmark)


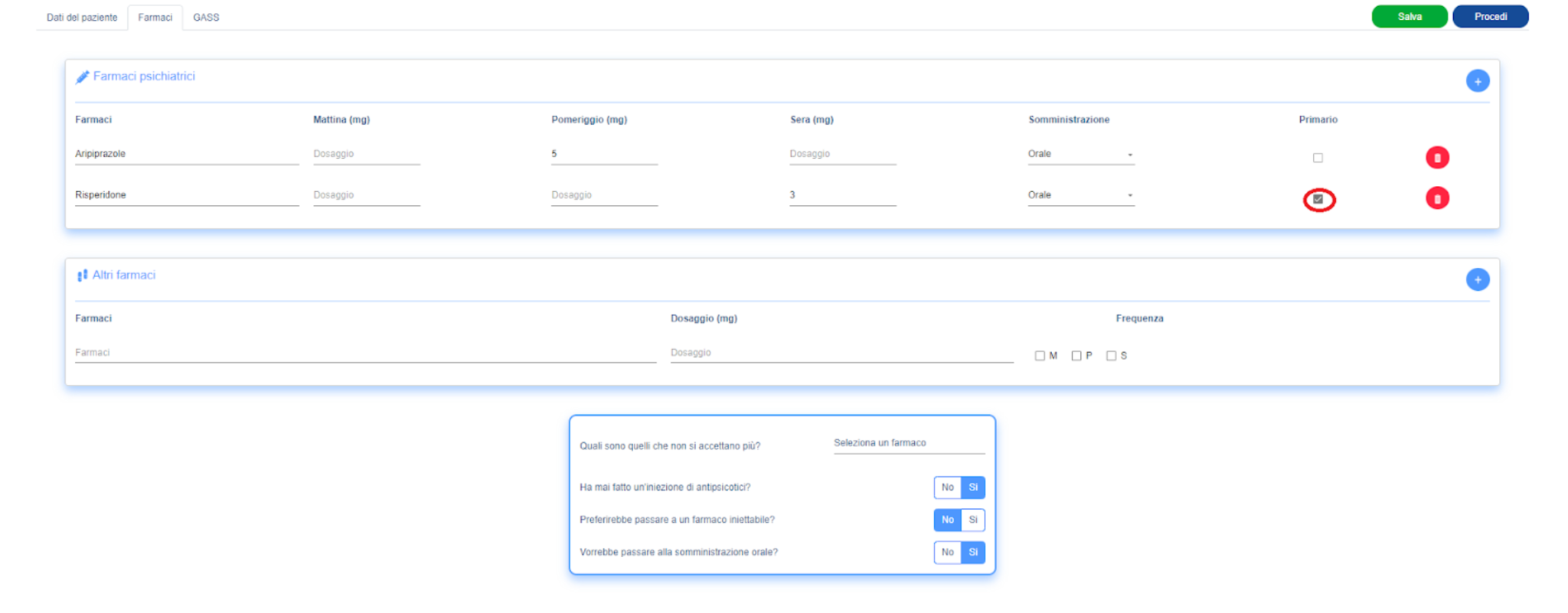


This screen allows clinicians to input psychiatric and other medications, specifying dosages, timing, and administration routes. The checkbox in the 'Primario' column designates the primary antipsychotic for future calculations.

# eAppendix img.4 - Side Effect Selection Interface


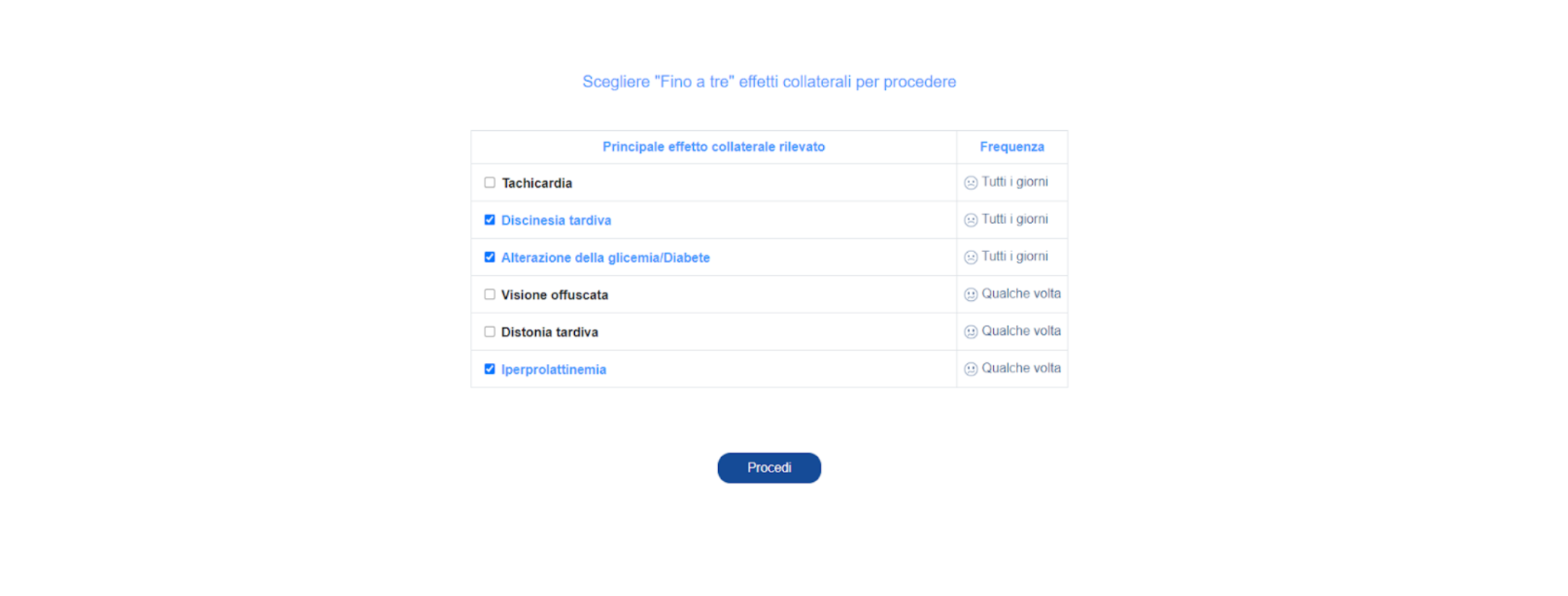


This screen allows patients to select up to three priority side effects from those identified in their Glasgow Antipsychotic Side Effects Scale assessment. The interface displays side effects with their frequency, enabling focused treatment planning.

# eAppendix img.5 - Side Effect Management Interface


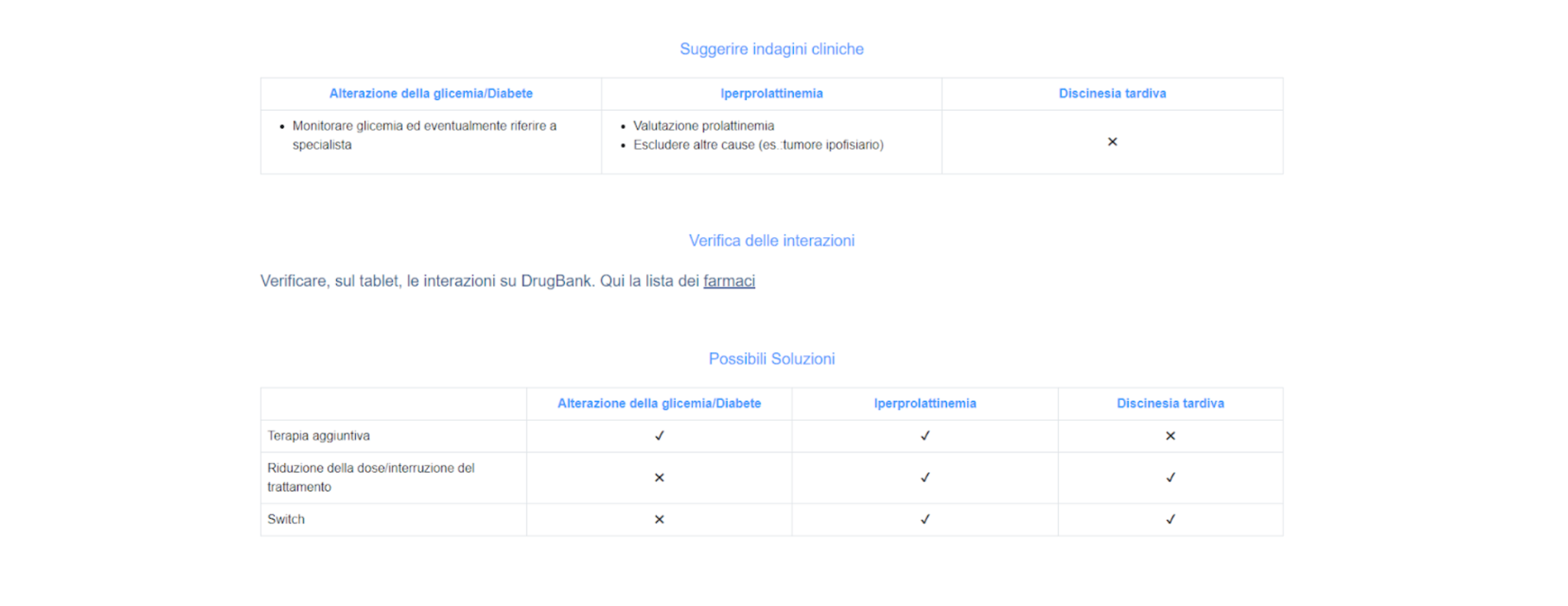


This screen displays recommended clinical investigations for selected side effects, provides a link to check drug interactions on DrugBank, and presents a matrix of potential solutions.

# eAppendix img.6 - Guideline-Based Treatment Options Matrix


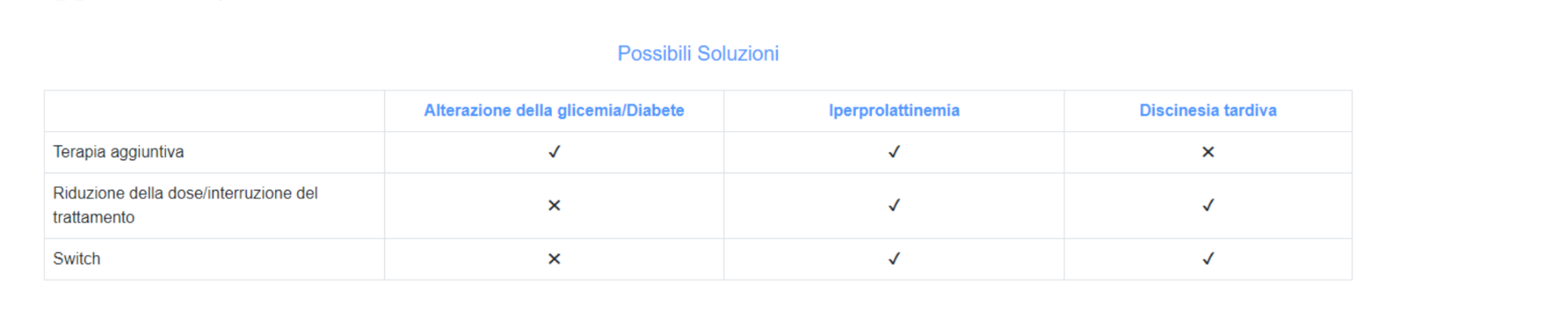


This matrix presents potential solutions for the three selected side effects. Checkmarks indicate interventions suggested by at least one guideline, while crosses denote non-recommended options. For comprehensive information on each option, refer to the main publication.

# eAppendix img.7 - Interactive Dose-Response Curve


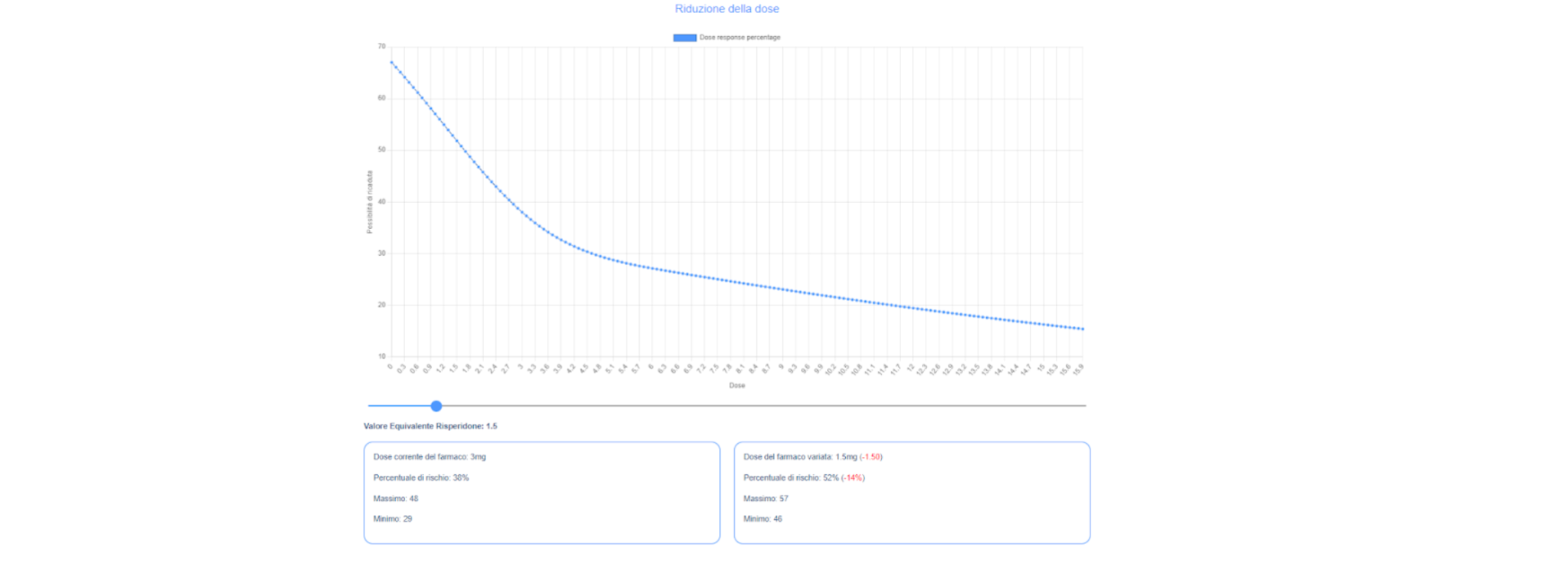


This graph displays the relationship between antipsychotic dose (x-axis) and relapse risk percentage (y-axis). An adjustable slider allows clinicians to explore different dosages and their corresponding relapse risks. The interface shows current and adjusted dose information, including equivalent Risperidone dosage, risk percentages, and minimum/maximum values, facilitating informed decision-making for dose adjustments.

# eAppendix img.8 - Forest Plot


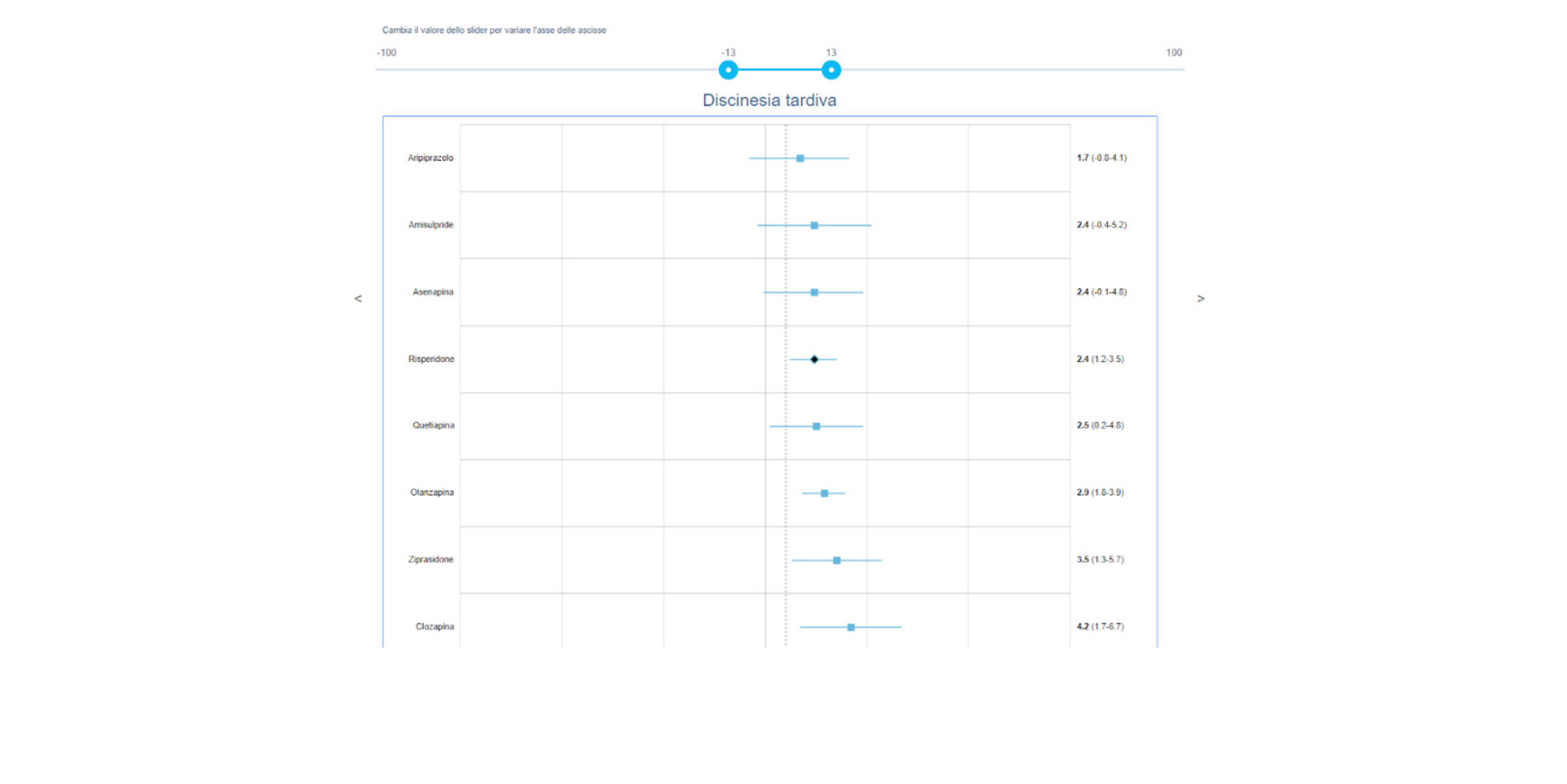


This forest plot compares the risk of a side effect among various antipsychotic medications. The plot displays odds ratios and confidence intervals for each drug, allowing for quick comparison of side effect risk. An adjustable slider at the top enables modification of the x-axis scale for detailed analysis.

# eAppendix Table 1 – Demographic characteristics of Physicians

| **ID** | **Age** | **Sex** | **Role** | **Work Place** | **Years of experience** |
| --- | --- | --- | --- | --- | --- |
| *Med01* | 52 | F | Specialist | Hospital | 27 |
| *Med02* | 36 | M | Specialist | Therapeutic Assisted Community | 5 |
| *Med03* | 50 | M | Specialist | Hospital | 24 |
| *Med04* | 32 | F | Resident | Hospital | 4 |
| *Med05* | 63 | F | Specialist | Hospital | 35 |
| *Med06* | 31 | M | Resident | Hospital | 2 |
| *Med07* | 30 | F | Resident | Hospital | 2 |
| *Med08* | 49 | F | Specialist | Hospital | 25 |
| *Med09* | 34 | M | Resident | Hospital | 1 |
| *Med10* | 31 | F | Resident | Hospital | 1 |
| *Med11* | 44 | M | Specialist | Mental health center | 15 |
| *Med12* | 63 | M | Specialist | Therapeutic Assisted Community | 30 |
| *Med13* | 30 | F | Resident | Hospital | 3 |
| *Med14* | 37 | F | Specialist | Mental health center | 10 |

# eAppendix Table 2 – Demographic charateristics of Patients

| **ID** | **Age** | **Sex** | **Medical facility** | **Education** | **Years of drug treatment** | **Number of psychotrpic medication assumed** | **Therapy changes (last 12 month)** | **Number of known non-psychiatric comorbidities** | **Number of different prescribers (other than general pratictioner)** |
| --- | --- | --- | --- | --- | --- | --- | --- | --- | --- |
| Pt01 | 27 | M | Hospital | High School | 1 | 1 | 0 | 1-2 | 1 |
| Pt02 | 30 | F | Hospital | University | 7 | 2-3 | 1-2 | 0 | 1 |
| Pt03 | 53 | F | Hospital | Middle School | 1 | 2-3 | 0 | 0 | 1 |
| Pt04 | 37 | M | Hospital | High School | 7 | 1 | 0 | 0 | 1 |
| Pt05 | 30 | M | Hospital | High School | 1 | 1 | 0 | 0 | 1 |
| Pt06 | 60 | M | Hospital | Middle School | 20 | 1 | 1-2 | 3-5 | 3-5 |
| Pt07 | 33 | M | Therapeutic Assisted Community | High School | 13 | 4-5 | 1-2 | 0 | 2 |
| Pt08 | 50 | M | Therapeutic Assisted Community | Primary School | 32 | 2-3 | 0 | 6-10 | 3-5 |
| Pt09 | 33 | M | Hospital | High School | 6 | 2-3 | 0 | 0 | 3-5 |
| Pt10 | 42 | M | Hospital | High School | 4 | 4-5 | 1-2 | 0 | 3-5 |
| Pt11 | 57 | M | Hospital | Middle School | 5 | 2-3 | 0 | 0 | 2 |
| Pt12 | 27 | M | Hospital | High School | 3 | 2-3 | 1-2 | 0 | 1 |
| Pt13 | 27 | F | Hospital | High School | 2 | 4-5 | 1-2 | 0 | 2 |
| Pt14 | 44 | F | Therapeutic Assisted Community | High School | 14 | 1 | 0 | 0 | 2 |
| Pt15 | 39 | M | Therapeutic Assisted Community | Middle School | 19 | 4-5 | 3-5 | 0 | 2 |
| Pt16 | 45 | M | Therapeutic Assisted Community | High School | 17 | 2-3 | 0 | 3-5 | 3-5 |

# eAppendix Table 3 – Physician’s Experience in Treating Mental and Psychotic Disorders and Opinions on the Use of Decision Aid Tools

| **Metric** | **Absolute Value** | **Percentage** |
| --- | --- | --- |
| Average Number of Schizophrenia Patients Treated per Month | 22.64 patients | N/A |
| Experience in Treating Mental Illness | Medium Experience: 5, Little Experience: 5, Much Experience: 5 | Medium Experience: 35.71%, Little Experience: 28.57%, Much Experience: 35.71% |
| Experience in Treating Psychotic Disorders | Much Experience: 6, Little Experience: 4, Medium Experience: 5 | Much Experience: 42.86%, Little Experience: 21.43%, Medium Experience: 35.71% |
| Belief in Improvement of Decision Quality by Decision Aid Tools | Yes: 13, Not Sure: 1 | Yes: 92.86%, Not Sure: 7.14% |
| Belief in Improvement of Communication by Decision Aid Tools | Yes: 10, Not Sure: 4 | Yes: 71.43%, Not Sure: 28.57% |

# eAppendix Table 4 – Patients’ Personal Use of Digital Devices and Internet and Knowledge of Medication Indications

| **Metric** | **Absolute Value** | **Percentage** |
| --- | --- | --- |
| Average Experience with Digital Devices (1-10) | 6.44 | N/A |
| Hours per Week Using Electronic Devices Privately | 8-14 hours: 6, 0-7 hours: 4, More than 28 hours: 4, 15-28 hours: 2 | 8-14 hours: 37.5%, 0-7 hours: 25.0%, More than 28 hours: 25.0%, 15-28 hours: 12.5% |
| Hours per Week Using Internet Privately | 0-7 hours: 5, More than 28 hours: 4, 8-14 hours: 3, 15-28 hours: 2 | 0-7 hours: 31.25%, More than 28 hours: 25.0%, 8-14 hours: 18.75%, 15-28 hours: 12.5% |
| Knowledge About Medication Indications | Know the indication of all my drugs: 13, I don't know: 1 | Know the indication of all my drugs: 92.86%, I don't know: 7.14% |

# eAppendix Table 5 – Participants relevant quotes extracted from the semi-structured interviews

**Dynamics of the Doctor-Patient Relationship and Involvement in Decisions**

| **Theme** | **Respondent ID** | **Quote** |
| --- | --- | --- |
| **Patients' perspectives** | | |
| Diverse attitudes towards patient involvement in decision-making | Pt08 | [I appreciate]"The opportunity to be involved in the decision-making process." |
| Strong deference to medical authority among patients desiring involvement | Pt10 | "Confidently, I rely on the doctor [to make the best decisions for my health]." |
|  | Pt11 | "If the doctor gives you the right medicine, you should take it." |
| Diverse attitudes towards patient communication with clinicians | Pt06 | "If the doctor asks me to come, for example, to a meeting, I might go [because I trust their judgment]." |
| Patients' satisfaction with involvement despite limited communication | Pt14 | "I feel cared for, not just treated as a number, and this possibility is beautiful." |
| **Clinicians' Perspectives** | | |
| 1) Clinician's prescriptive approach and paternalistic tendencies  2) Patient needs and engagement in decision-making post-stabilization | Med01 | "We try [to involve patients in their treatment plans] even if we don't always succeed because it depends on the patient's awareness." |
| Importance of patient insight in shared decision-making | Med11 | "My experience always leads me to distinguish between the patient's awareness, their level of cooperation, and so on..." |
| Concerns about information overload and misinterpretation | Med11 | "Often, the request [for more information] is also underpinned by a non-acceptance of the therapy and sometimes may even conceal a more or less hidden desire to stop taking the therapy altogether [which can be challenging to manage]." |
| Insights of patients’ attitude on independently searched information | Med02 | "[The information patients find online is] often irrelevant [to their specific conditions or treatment plans]." |
|  | Med14 | "If they are young, often, let's say under 50 years old, very often, also because I ask them [about their research]. Above 50 years, they either don't do it or rely on word of mouth, or if they do, they are, let's say, shy to talk about it, so it's up to me to draw it out of them [to ensure they receive accurate information]." |
|  | Med13 | "It depends on the patient […] in the sense that they actually do not share all the concerns they may have developed from informing themselves through the Internet [which can limit our understanding of their fears and misinformation]." |

**The App in the Management of Treatment and Side Effects**

| **Theme** | **Respondent ID** | **Quote** |
| --- | --- | --- |
| **Patients' Perspectives** | | |
| Varied views on the utility of STEP-SE for improving trust in medical decisions | Pt02 | "It improves the doctor’s life, who has to find a solution. It improves compliance if it’s used between the doctor and the patient. [...] I know from the chart that clozapine is better compared to aripiprazole, which I no longer use, or paliperidone, meaning I don’t have to try 3000 more drugs." |
|  | Pt06 | "Satisfied with their doctor relationship, doubted the app's additional benefits." |
| App as a potential part of therapy and its role in enhancing the therapeutic alliance | Pt14 | "In the app, I like this immediate doctor-patient confrontation; it brings us even closer than a regular visit." |
| App’s role in patient autonomy and motivation | Pt04 | "The app helps [me] understand the importance of using certain medications when one has had a health problem or still has it." |
|  | Pt05 | "I wouldn’t be afraid to use the medication." |
| Concerns about potential misunderstandings arising from app use | Pt16 | "It can lead to misunderstandings because, for example, I would like to decrease the olanzapine, but the psychiatrist says it should not be reduced." |
| **Clinicians' Perspectives** | | |
| App as a tool for enhancing patient involvement and therapeutic choice-making | Med03 | "I will continue to trust my experience [...]. However, if I gradually see that it tells me something different from what I know, I will also start to try it and possibly change my mind." |
| Efficiency in identifying side effects and formulating treatment strategies | Med07 | "It saves the patient from searching for information that could confuse them, therefore providing them with a clear guide and knowing that they should not rely solely on the professional's directive, but that the professional is basing their decisions on studies, so the trust is there both from a human perspective and from a scientific one." |
| Appreciation of the app as a reliable source of information and SDM tool | Med14 | "If there are interactions between drugs [...] I could show them physically, make [patients] feel involved and make their symptoms feel important, so trust would play a big role; it would increase mutual trust." |
| Concerns about potential information overload and misinterpretation risks | Med01 | "Warned of potential information overload for patients." |
|  | Med09 | "Cautious about content that may alarm patients unnecessarily." |
|  | Med13 | "Pointed out risks of misinterpretation or excessive reliance on the app's suggestions for medication changes." |
|  | Med11 | "I would select the use of this app for patients I know better, where shared decisions can truly improve clinical outcomes, without the risk of setbacks." |

# Semi-structured interview – Patients

1. How often do you talk to your doctor about health information you have researched on your own?
2. How do you feel when discussing possible therapies with your doctor?
3. In your experience, how are decisions about future treatments made? Does this also apply to decisions about your mental illness?
4. Where do you see room for improvement in how you can participate in treatment decisions? What would help you make better decisions?
5. Describe your experience in learning to use STEP-SE. Which parts did you find easier or more difficult? Did the initial information help you understand how to use it?
6. You were shown two different strategies for presenting information on changing antipsychotics: one through graphs and the other through a descriptive table. Which format did you find easier to understand, and which would you feel more comfortable using to make a decision? Could you explain your answer by providing examples of pros and cons of each?
7. How do you think STEP-SE allows you to manage antipsychotic-induced side effects more practically?
8. Are there any steps or processes in STEP-SE that you find unnecessarily complicated or lengthy? How do you think they could be simplified?
9. If you could change one thing in STEP-SE to make it easier to use, what would it be and why?
10. Do you think you would be able to use STEP-SE even after a period of non-use?
11. What problems and/or errors have you encountered while using STEP-SE? If you found any, how did you resolve them? Was it easy to resolve?
12. Are there parts of STEP-SE that you think could easily lead to errors or misunderstandings? If so, what are they and why?
13. What do you like most about STEP-SE and why?
14. What do you like least about STEP-SE and why?
15. Are you satisfied with the functionalities offered by STEP-SE? Why? What would you add?
16. If you could change something about the interface and graphics of STEP-SE, what would it be and why?
17. How do you think the use of STEP-SE has affected your doctor/patient relationship?
18. Do you think the use of STEP-SE has affected your psychological well-being? How?
19. How has STEP-SE influenced your sense of control or autonomy in managing your health?
20. Do you believe the use of STEP-SE has had an impact on your motivation or commitment to treatment? If so, how and why?

# Semi-structured interview – Clinicians

1. To what extent do patients discuss with you health information they have researched on their own?
2. How do patients perceive the discussion about treatment options?
3. In clinical experience, how are decisions about future treatments made? Does this also apply to treatment decisions related to mental disorders?
4. What are the opportunities to improve patient participation in therapeutic decisions? What tools or methods could facilitate the decision-making process?
5. Describe your experience in learning to use STEP-SE. Which parts did you find easier or more difficult? Did the initial information help you understand how to use it?
6. You were shown two different strategies for presenting information on changing antipsychotics: one through graphs and the other through a descriptive table. Which format did you find easier to understand, and which would you feel more comfortable using to make a decision? Could you explain your answer by providing examples of pros and cons of each?
7. How do you think STEP-SE enables you to manage antipsychotic-induced side effects more practically?
8. Are there any steps or processes in STEP-SE that you find unnecessarily complicated or lengthy? How do you think they could be simplified?
9. If you could change one thing in STEP-SE to make it easier to use, what would it be and why?
10. How do you think STEP-SE affects your daily workflow? Does it help you save time or do more things?
11. Do you think you would be able to use STEP-SE even after a period of non-use?
12. What problems and/or errors have you encountered while using STEP-SE? If you found any, how did you resolve them? Was it easy to resolve?
13. Are there parts of STEP-SE that you think could easily lead to errors or misunderstandings? If so, what are they and why?
14. What do you like most about STEP-SE and why?
15. What do you like least about STEP-SE and why?
16. Are you satisfied with the functionalities offered by STEP-SE? Why? What would you add?
17. If you could change something about the interface and graphics of STEP-SE, what would it be and why?
18. How do you think the use of STEP-SE has affected your doctor/patient relationship?
19. Do you think the use of STEP-SE has affected your psychological well-being? How?
